# Supplementary material for: The Use of Scoring Hip Osteoarthritis with MRI as an Assessment Tool for Physiotherapeutic Treatment in Patients with Osteoarthritis of the Hip
Source: J Clin Med. 2021 Dec 21;11(1):17. doi: 10.3390/jcm11010017 (PMC8745579; doi:10.3390/jcm11010017)
Supplement: Supplementary file 1 [file jcm-11-00017-s001.zip › Table S1. The average time required to score both joints.pdf]

Table S1. The average time required to score both joints

Reading	Scoring time	
	**P.P.**	**K.P.**
Baseline 1	21 min 15 s ± 3 min 22 s	24 min 10 s ± 8 min 22 s
Baseline 2	22 min 30 s ± 5 min 42 s	18 min 17 s ± 6 min 30 s
Follow-up	17 min 8s ± 3min 16s	15 min 12s ± 3 min 33s
