# Supplementary figures and images for: The Use of Scoring Hip Osteoarthritis with MRI as an Assessment Tool for Physiotherapeutic Treatment in Patients with Osteoarthritis of the Hip
Source: J Clin Med. 2021 Dec 21;11(1):17. doi: 10.3390/jcm11010017 (PMC8745579; doi:10.3390/jcm11010017)

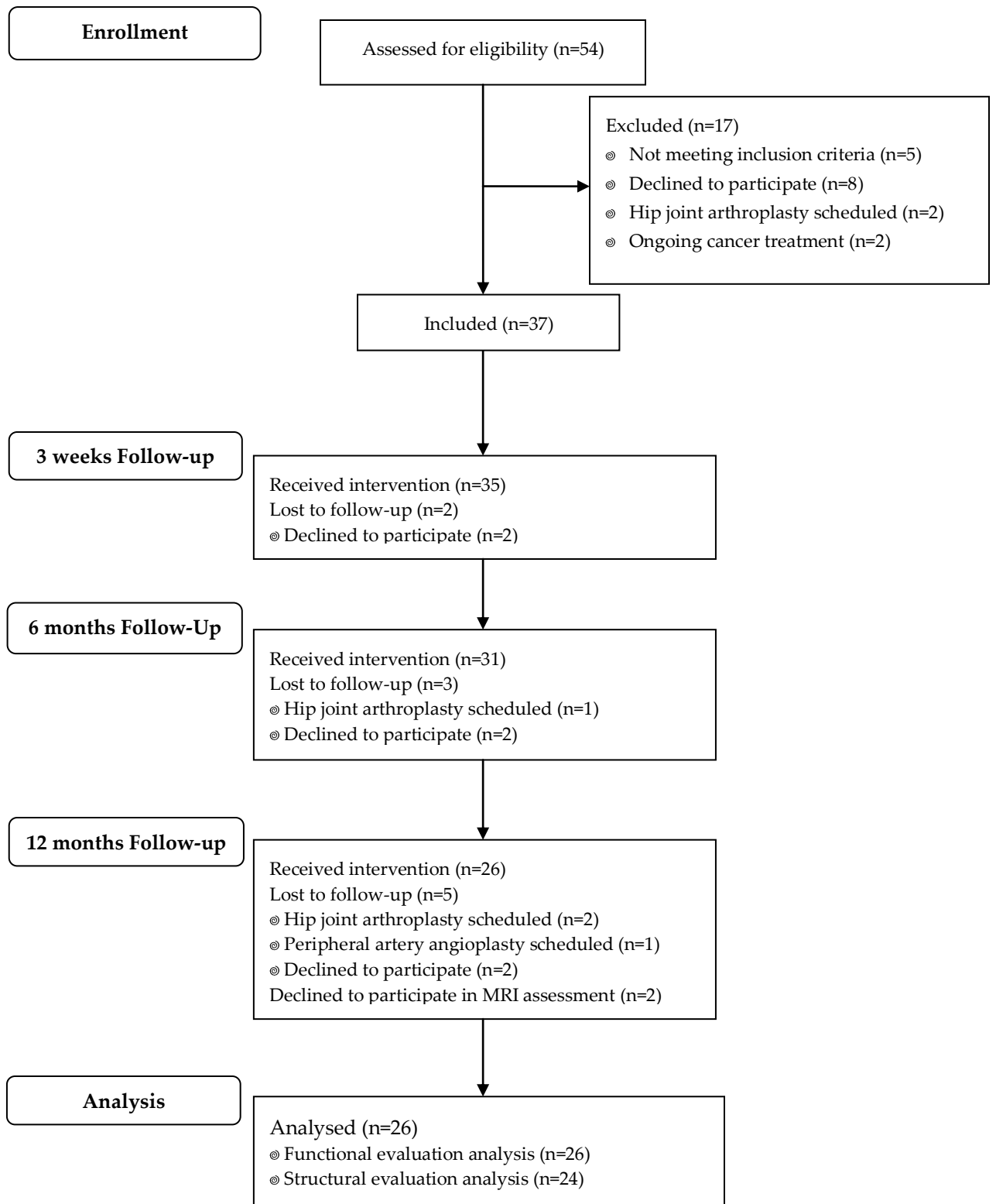

**Figure S1.** Patients' enrolment and completion

Supplement: Supplementary file 1 [file jcm-11-00017-s001.zip › Figure S1. Patients enrolment and completion.pdf]
